# Gene expression profiling of human alveolar macrophages infected by *B. anthracis *spores demonstrates TNF-α and NF-κb are key components of the innate immune response to the pathogen

**DOI:** 10.1186/1471-2334-9-152

**Published:** 2009-09-10

**Authors:** Mikhail Dozmorov, Wenxin Wu, Kaushik Chakrabarty, J Leland Booth, Robert E Hurst, K Mark Coggeshall, Jordan P Metcalf

**Affiliations:** 1 Department of Urology, University of Oklahoma Health Sciences Center, Oklahoma City, OK, USA; 2 Department of Biochemistry and Molecular Biology, University of Oklahoma Health Sciences Center, Oklahoma City, OK, USA; 3 Pulmonary and Critical Care Division, Department of Medicine, University of Oklahoma Health Sciences Center, Oklahoma City, OK, USA; 4 Program in Immunobiology and Cancer, Oklahoma Medical Research Foundation, Oklahoma City, OK, USA; 5 Department of Microbiology and Immunology, University of Oklahoma Health Sciences Center, Oklahoma City, OK, USA

## Abstract

**Background:**

*Bacillus anthracis*, the etiologic agent of anthrax, has recently been used as an agent of bioterrorism. The innate immune system initially appears to contain the pathogen at the site of entry. Because the human alveolar macrophage (HAM) plays a key role in lung innate immune responses, studying the HAM response to *B. anthracis *is important in understanding the pathogenesis of the pulmonary form of this disease.

**Methods:**

In this paper, the transcriptional profile of *B. anthracis *spore-treated HAM was compared with that of mock-infected cells, and differentially expressed genes were identified by Affymetrix microarray analysis. A portion of the results were verified by Luminex protein analysis.

**Results:**

The majority of genes modulated by spores were upregulated, and a lesser number were downregulated. The differentially expressed genes were subjected to Ingenuity Pathway analysis, the Database for Annotation, Visualization and Integrated Discovery (DAVID) analysis, the Promoter Analysis and Interaction Network Toolset (PAINT) and Oncomine analysis. Among the upregulated genes, we identified a group of chemokine ligand, apoptosis, and, interestingly, keratin filament genes. Central hubs regulating the activated genes were TNF-α, NF-κB and their ligands/receptors. In addition to TNF-α, a broad range of cytokines was induced, and this was confirmed at the level of translation by Luminex multiplex protein analysis. PAINT analysis revealed that many of the genes affected by spores contain the binding site for c-Rel, a member of the NF-κB family of transcription factors. Other transcription regulatory elements contained in many of the upregulated genes were c-Myb, CP2, Barbie Box, E2F and CRE-BP1. However, many of the genes are poorly annotated, indicating that they represent novel functions. Four of the genes most highly regulated by spores have only previously been associated with head and neck and lung carcinomas.

**Conclusion:**

The results demonstrate not only that TNF-α and NF-κb are key components of the innate immune response to the pathogen, but also that a large part of the mechanisms by which the alveolar macrophage responds to *B. anthracis *are still unknown as many of the genes involved are poorly annotated.

## Background

*Bacillus anthracis *is a gram-positive, aerobic, spore-forming, rod-shaped bacterium which causes a virulent disease, anthrax. The three primary forms of the disease are due to three different mechanisms of exposure: ingestion (gastrointestinal), contact (cutaneous) or inhalation (inhalational) [1]. Inhalational anthrax is the most life-threatening form of the disease [2,3], and was the type seen during the recent bioterrorism attacks [4].

Inhalational anthrax is characterized by a rather unique finding in that the inhaled spores do not vegetate and cause disease at the site of entry [5,6]. Instead, spores are rapidly and efficiently phagocytosed by alveolar macrophages and dendritic cells, and carried through lung tissue to the regional lymph nodes [5,7-9]. It is only after the pathogen escapes the lung that dissemination occurs, following transit to the thoracic lymph nodes. There is a significant delay, as long as 43 days, between exposure and clinical disease, implying that there is temporary containment of the pathogen, likely by the innate immune system [10].

Alveolar macrophages play a central role in the innate immune system and are the first line of defense against inhaled pathogens. They are the most prominent resident cells that not only engulf and kill infectious agents, but also produce numerous modulators of the inflammatory response to recruit and activate additional cells of the immune system. Alveolar macrophages also provide a link to the adaptive immune system since they function as antigen presenting cells. Previous studies of the transcriptional response of the murine macrophage-like RAW 264.7 cell line to *B. anthracis *(Sterne) spore infection provided initial insight into macrophage responses [11] and *B. anthracis *adaptation to the host microenvironment [12], but correlation of these results with human alveolar macrophage responses has not been performed.

Our earlier studies examined the interaction of *B. anthracis *spores with human alveolar macrophages (HAM) obtained by bronchoalveolar lavage (BAL). We studied the initial events after exposure to spores beginning with the rapid internalization of spores by the macrophages. Spore exposure rapidly activated the mitogen-activated protein kinase (MAPK) signaling pathways ERK, JNK, and p38. This was followed by transcriptional activation of cytokine and primarily monocyte chemokine genes and the data was confirmed at the level of translation [13].

In the current study, we infected the HAM obtained by BAL with *B. anthracis *(Sterne) spores and performed Affymetrix Human Genome U133 Plus 2.0 Array (Santa Clara, CA) to provide a comprehensive view of the innate immune response of the alveolar macrophage to the pathogen. In comparing the expression pattern of spore-exposed with mock-infected cells, our analysis identified TNF-α and NF-κB as key components of the innate immune response to *B. anthracis*. Many (48) of the genes affected by spores shared c-Rel transcription regulatory element, a member of NF-κB family of transcription factors. Our findings are consistent with our previous, more limited evaluation of the immune response by HAM to spores using ribonuclease protection assay (RPA) and ELISA [13]. In addition, our results show the actions of a significant number of poorly annotated genes, indicating that much of the response occurs due to currently unknown mechanisms.

This study is the first detailed microarray analysis to describe the HAM response to *B. anthracis *spores. It provides a tool for investigators to use for diagnostic and therapeutic purposes and points toward a number of unknown processes as being important in the macrophage response.

## Methods

### Preparation of *Bacillus anthracis *spores

*B. anthracis*, Sterne strain 7702 (pX01^+^, pX02^-^), was kindly provided by Dr. Jimmy Ballard (University of Oklahoma Health Sciences Center, Oklahoma City). Bacteria were grown overnight at 37°C with continuous shaking in Luria-Bertani broth (LB) media and were then streaked onto AK Agar sporulating slants. Bacteria were incubated for three weeks at 30°C. The slants were washed with 10 ml of chilled, sterile, deionized water and spun at 10,000 × g for 10 minutes and resuspended in 10 ml chilled water. The spore suspension was heated at 65°C degrees for 30 minutes to kill vegetative bacteria. After heat treatment the spores were centrifuged for 10 minutes at 10,000 × g. The supernatant and the very top layer of the pellet were aspirated and then the spores were resuspended in chilled sterile deionized water and centrifuged for 10 minutes. The pellet was washed 5 times to remove contaminating cell debris. The titer of the spore preparation was determined by plate counts. The spores were diluted to 1 × 10^9 ^spores/ml and stored at 4°C. Titers were reconfirmed by plate counts before each use. There was no detectable endotoxin in the final spore dilutions used in the experiments as determined by limulus amebocyte lysate assay (Cambrex, Walkersville, MD).

### Collection of human alveolar macrophages

Macrophages were obtained by bronchoscopy with the consent of human subjects following a protocol approved by the Oklahoma University Health Sciences Center Institutional Review Board and the Institutional Biosafety Committee. These volunteers were healthy subjects with no smoking history or history of pulmonary disease. Cells were collected in sterile saline solution from human subjects and centrifuged at 500 × g for five minutes. The supernatant was removed and the pellets were washed in 10 ml of RPMI-1640 containing 50 μg/ml gentamicin and resuspended in 10 ml RPMI + 2% fetal calf serum (FCS) with 50 μg/ml gentamicin. Cell counts were determined by a hemocytometer; cell type was determined by morphology using Diff-Quick staining (Baxter, Miami, FL); and cells were resuspended to a concentration of 1 × 10^6 ^macrophages/ml. There were >95% macrophages in each cell preparation. One ml of cells per well was plated into 24 well plates and allowed to incubate for 2 to 4 h to facilitate attachment. Subsequently, the media was removed and fresh media containing 2% FCS and gentamicin was added. The cells were incubated overnight at 37°C with 5% CO_2_.

### Infection and RNA isolation

Human alveolar macrophages were plated into six well culture plates at 1 × 10^6 ^cells/ml in RPMI with 2% FCS containing 50 μg/ml of gentamicin and, following overnight attachment, were stimulated with *B. anthracis *spores (1 MOI) for 6 h. This time was chosen, based on previous experiments showing that peak cytokine mRNA induction by spores occurred between 5 and 7 hours after infection [14]. Unstimulated control wells were prepared by exposing cells to equal volumes of spore diluent (sterile distilled water). Cells were harvested by addition of TRIzol reagent (Invitrogen), and total RNA was isolated according to the manufacturer's protocol using glycogen (20 mg/ml) as the carrier. Cells yielded 8-10 μg total RNA/well. Gentamicin was present throughout the experiment. This was done to ensure the responses measured were to the spores, and not to vegetative bacteria. Vegetative bacteria are very sensitive to Gentamicin, and the amount of gentamicin used (50 μg/ml) is the standard amount used for culture of alveolar macrophages. We have not found that this concentration of gentamicin affects macrophage gene expression. However, it does prevent production of any *B. anthracis *vegetative bacteria as determined by microscopy, or any biologically active bacterial virulence toxins.

### RNA extraction, cRNA preparation, array hybridization & scanning

An RNA cleanup step was performed using Qiagen's RNeasy kit. First- and second-strand cDNA synthesis was carried out with 5 μg total RNA as starting material (SuperScript™ cDNA Synthesis kit, Invitrogen). Biotin-labelled cRNA was prepared using the Affymetrix RNA Transcript Labelling kit, and fragmented in Fragmentation Buffer (Affymetrix). Labelled, fragmented cRNA was hybridized to GeneChip Human Genome U133 Plus 2.0 arrays (Affymetrix), which were then washed, stained and scanned as described [15]. The HG-U133 Plus 2.0 arrays comprised >54,000 probe sets representing >47,000 transcripts and variants, including 38,500 well-annotated genes. Four microarrays were used for each experimental condition, however, quality control revealed poor quality of one microarray in the test (spore infected) condition. Therefore, four and three arrays were used in the control and test conditions, respectively.

### Microarray Data analysis

**Figure 1:**
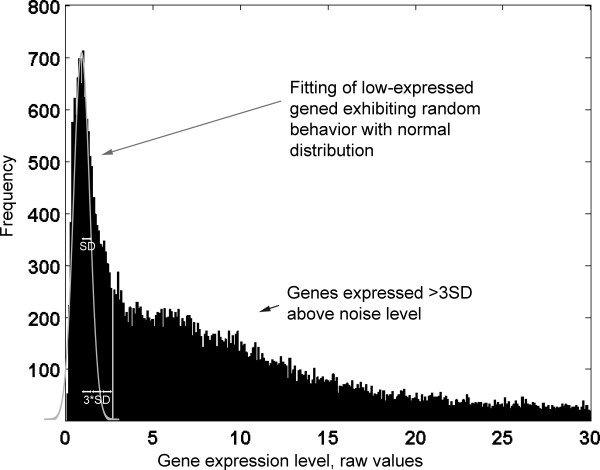
**Frequency histogram of gene expression in one microarray**. The X-axis shows the raw gene expression level, cut at 30 for clarity. The Y-axis shows the number of genes at a given expression level. The white line outlines a normal distribution fit to low-expressed genes exhibiting random behavior.

HG-U133 Plus 2.0 array data for each experimental condition were exported from the Affymetrix Suite Software for further analysis. The data were normalized as described previously [16,17], using the variability of low expressed genes as a reference point. In order to find genes expressed above the level of technical noise, a frequency histogram of raw expression values was examined for each array. The histogram yielded a right-skewed unimodal distribution curve with the mode around 2 (Figure 1). A normal distribution curve representing the variability of the data around zero was then fitted around the mode, mirroring the Gaussian profile of the left part of the histogram. Its parameters were then defined (mean, SD) and the data were normalized to the standard deviation of the noise after subtraction of the mean. The arrays were then Log10-transformed and adjusted to each other by robust linear regression under the assumption that the expression of most genes does not change. The data were then filtered to remove genes with an expression value less than 3.0. This is equivalent to setting a threshold at 3 SD above the noise level. Genes expressed below the noise level under all experimental conditions (about 1,600) were excluded from consideration, as their expression cannot be reliably assessed. Full microarray data were deposited in the Gene Expression Omnibus (GEO) and are accessible on the GEO web-site (GSE14390).

To identify differentially expressed genes between two experimental groups, we used the associative analysis as described by Dozmorov *et al*. [18]. Briefly, in pooled microarray datasets, a reference group of genes expressed above background with low variability of expression was identified by an F-test. The assumption is that because most genes do not change expression in any experiment, this variability of expression among this group is due to random, technical factors alone. Genes with statistically significantly variability above the random, technical variation are assumed to vary for biological reasons. A *t*-test termed "associative *t*-test" was applied to test if a given gene belongs to or differs from this group. The associative *t*-test is a standard *t*-test applied to the comparison of expression variability rather than differences in mean values. To identify an evaluable number of differentially expressed genes, a stringent criterion was applied. Only genes expressed below noise level in one group and greater than 10 SD above noise level in another group were selected for further analysis. For genes that were expressed above noise level in both conditions, genes that have greater than 2-fold differences in expression levels and expressed greater than 20 SD above noise level in at least in one condition were also selected. These genes can be considered to be "beacons" that point to pathways or gene networks that are altered.

### Identification of significantly overrepresented functions, networks and canonical pathways

Lists of genes from individual clusters were submitted to Ingenuity Pathway Analysis (IPA; Ingenuity^® ^Systems, Redwood City, CA, http://www.ingenuity.com). Ingenuity maps gene IDs to its database and performs statistical computing to identify the most significant ontologies, canonical pathways, and networks overrepresented in a given gene list as compared with the whole list of genes in the Human Genome U133 Plus 2.0 array. By default p < 0.05 was used in all calculations. Gene lists from each group were analyzed for overrepresented general functions, canonical pathways, and the networks that can be assembled from them.

To classify each cluster in more detail by their ontological properties, we used the Database for Annotation, Visualization and Integrated Discovery (DAVID) [19]. The Gene Functional Classification tool in DAVID builds clusters of genes with significantly similar ontologies as tested against the whole list of genes in the Affymetrix Human Genome U133 Plus 2.0 array. Medium stringency was used to yield a comprehensive set of ontological groups and to group genes with similar functions. Increasing or decreasing stringency resulted in identification of fewer or more groups of genes with similar functions but did not produce any additional information.

### Analysis of common transcription regulatory networks

The Promoter Analysis and Interaction Network Toolset (PAINT, v 3.6) identifies common upstream transcription factor binding sequences in a list of genes and compares the prevalence of sequences within the set to that expected to be found by chance in a given list of transcription regulatory elements (TREs) http://www.dbi.tju.edu/dbi/tools/paint/[20,21]. Important features of PAINT include that it is independent of expression level, being a function of sequence only, and that it searches for overrepresented TREs in upstream sequences only. Each group of genes was tested for significantly overrepresented TREs against the whole set of genes in the Human Genome U133 Plus 2.0 array, which represent a universe of all TREs affected by the experimental conditions used. The default set of parameters was used for the analysis, i.e. organism - Homo Sapiens, desired upstream length - 2000 bp. MATCH (Transfac Public) database was used to retrieve promoter sequences, with filter option - minimize false positives, core similarity threshold equal to 1, and "find TREs on complimentary strand" checkbox checked. Genes and TREs were clustered according to their default settings. Statistical significance for TRE overrepresentation was set as p < 0.05 with additional filtering performed by setting the false discovery rate (FDR) at the level described (see Results section).

### Luminex Protein Array

Human alveolar macrophages were plated into six well culture plates at 1 × 10^6 ^cells/ml in RPMI with 2% FCS containing 50 μg/ml of gentamicin and, following overnight attachment, the media for all cells was replaced with identical media containing gentamicin and some cells were stimulated with *B. anthracis *spores (1 MOI) for 7.5 h. Unstimulated control wells were prepared by exposing cells to equal volumes of spore diluent (sterile distilled water). Supernatants were collected and subjected to Multiplex Luminex Protein Array in the Luminex Core Facility of Baylor Institute for Immunology Research (Dallas, TX).

### Oncomine data analysis

Oncomine [22,23] is a cancer microarray repository, containing >18,000 gene expression profiles from a diverse number of microarray studies. This repository was used for this study to obtain some information on the lung-specific expression of some of the poorly annotated genes. Its web-based interface allows users to queue genes of interest and determine their differential and/or co-expression patterns among different diseases, stages of diseases and responses to a particular treatment. Affymetrix probe ID numbers of unknown genes were entered into the "Gene search" field. The conditions in which those genes were overrepresented were identified by Oncomine meta-analysis. Most significant conditions (i.e. with smallest p-values) were selected.

## Results

### Differentially expressed genes were predominantly upregulated by *B. anthracis *spores

Two sets of differentially expressed genes between mock treated (control) and spore infected (test) conditions were identified (Table 1). The first set of genes identified were those whose raw expression was more than 20 standard deviations above noise level in at least in one experimental condition and also had at least a two-fold induction of expression over the other condition (mock or spore treated). In the second set, genes were identified that were expressed under one experimental condition or the other. These genes were defined as being expressed under noise level in one of the experimental conditions, and in the other condition, they were expressed at least 10 standard deviations above noise level. In the first set, the majority (209 unique identifiers) were overexpressed in cells infected with spores while only 61 genes were down-regulated in infected cells (expressed at higher levels in mock infected cells). In the second set, 43 genes were expressed only in spore infected cells and 21 genes were expressed only in mock infected cells.

**Table 1:** Proportion of genes expressed under different conditions.

Genes upregulated in spore infected HAM	Overexpressed genes (1^st ^set)	209
	Expressed only in spore-treated (2^nd ^set)	43
	Total upregulated	252
Genes downregulated in spore infected HAM	Downregulated genes (1^st ^set)	61
	Expressed only in mock-treated (2^nd ^set)	21
	Total downregulated	82

Genes overexpressed (the first set) and genes uniquely expressed in a given condition (the second set) were combined for further analysis (Table 1). A total of 252 genes were identified as upregulated and 82 genes were downregulated in spore infected HAMs. It should be noted that those numbers reflect the number of unique identifiers on HG-133 Plus 2 Affymetrix arrays. Gene annotations, however, can overlap with different Affymetrix IDs (i.e. 209201_x_at and 217028_at both identify chemokine (C-X-C motif) receptor 4 CXCR4, with the former probe set related to multiple transcripts and the latter to a unique transcript for this gene [24]). The full list of differentially expressed genes is shown in Additional File 1.

### Apoptosis, Immune response and inflammation genes are induced by *B. anthracis *spores

Functional classification was performed by using Ingenuity Pathway analysis, which identifies general functions in a list of genes, and DAVID, which classifies genes by their ontological groups. It should be noted that interpreting the pathways and functions of a set of differentially expressed genes can only assess genes of known functions operating by known mechanisms. Often these well-annotated genes represent a minority of genes identified. Because half the genome is poorly annotated [25], the truly new knowledge arising from these experiments resides in the functions of the poorly annotated genes. That is the case here because of the 252 genes identified, only 126 are sufficiently well annotated for pathway analysis by Ingenuity Pathway Analysis. The fact that known pathways or networks are found gives credence to the identification of differentially expressed but unknown genes as being important. The general functions among 139 genes mapped by Ingenuity out of a total of 252 spore-upregulated genes were Cell Death (68 genes, p < 1.8 × 10^-15^), Cellular Growth and Proliferation (62 genes, p < 5.9 × 10^-15^), Hematological disease (39 genes, p < 1.25 × 10^-14^) and Immunological Disease (49 genes, p < 1.25 × 10^-14^).

Under medium stringency DAVID clustered these genes into eight functional groups. The first and most statistically significant cluster contained 7 genes related to Apoptosis. Only unique gene names are counted, since the gene can be recognized by multiple Affymetrix IDs (see above). Included in this group is one member of the TNF superfamily TNFAIP8 and the immediate early response 3 (IER3) gene. Five chemokine ligands comprised the second cluster, and upregulation of transcription was the common function of the 35 genes in the third cluster. Genes responsible for transcriptional activation were also annotated as being related to immunological functions, and included HIVEP2 (human immunodeficiency virus type I enhancer binding protein 2), NFATC1 (nuclear factor of activated T-cells, cytoplasmic, calcineurin-dependent 1). Interestingly, the fourth cluster contained 2 keratin associated genes. The remaining clusters were zinc finger, protein phosphatase, and transmembrane proteins and receptors (Table 2).

**Table 2:** Ontological clustering of genes upregulated on spore infected cells.^1^

Cluster	No. of genes	Annotated genes	Ontology
1	8	BRE, DDIT4, IER3, MCL1, PPP1R15A, SERPINB9, TNFAIP8	Apoptosis
2	8	CCL20, CCL3L1, CCL4L1, CCL5, CXCL2	Chemokine activity
3	37	CXORF43, EGR1, ENO3, ERF, EZH2, FALZ, FLJ25169, FOSL1, GLIS2, HEY1, HIC2, HIVEP2, KLF12, KLF5, LHX3, LOC400713, MYBBP1A, NA, NFATC1, NFE2L2, NR4A2, NR4A3, PCGF3, PNRC1, PROP1, PRRX1, REST, SKIL, THRAP1, ZBTB10, ZFHX4, ZFX, ZNF11B, ZNF586, ZNF587	Transcription
4	4	KRTAP5-5, TCHP	Keratin filament
5	8	IBRDC3, MNAB, PCGF3, REST, RNF17, TAB3, TEX13A, ZNF586	Zinc-finger
6	4	DUSP1, DUSP16, DUSP2, UBLCP1	Protein phosphatase
7	16	BTNL9, CD44, CD6, CXCR6, DRD2, F3, GP1BB, GPR84, GYPA, ICAM1, PCDHA9, PUNC, PVR, SLAMF7, TBXA2R, TNFSF9	Transmembrane
8	4	FAM11A, FLJ37478, MFSD2, REEP5	Transmembrane

^1 ^Only unique gene names shown in "Annotated genes" column since multiple Affymetrix IDs can map to the same gene name.

The overall picture was less clear with regards to genes downregulated in spore infected cells. Out of the 82 downregulated genes, Ingenuity could map only 35 as eligible for functions or pathways. These genes have been associated with very generic ontologies, such as Gene Expression (5 genes, p < 1.89 × 10^-4^) and Protein Trafficking (2 genes,1.89 × 10^-4^). Several remarkable players include thrombospondin (THSD4), phosphatidylinositol-4-phosphate 5 (PIP5KL1) and phosphoinositide-3 (PIK3C2B), frizzled homolog 2 (FZD2), erythropoietin receptor (EPOR), sry box 4 (SOX4), chemokine (c-x-c motif) receptor 4 (CXCR4), interleukin 17 receptor b (IL-17RB), immediate early response 5-like (IER5L), and YWHAZ. The full list of these genes is shown in Additional File 1.

DAVID analysis did not bring more light to the picture, yielding only three ontological clusters under medium stringency. The first cluster contained 7 genes related to zinc ion binding, and there were 15 genes in the second cluster (RNA metabolism), many of which were also zinc finger proteins. The third cluster contained 4 transmembrane receptors. Full ontological tables including Affymetrix IDs are shown in Additional File 2.

### TNF-α and NF-κB upregulation in response to *B. anthracis *spores

**Figure 2:**
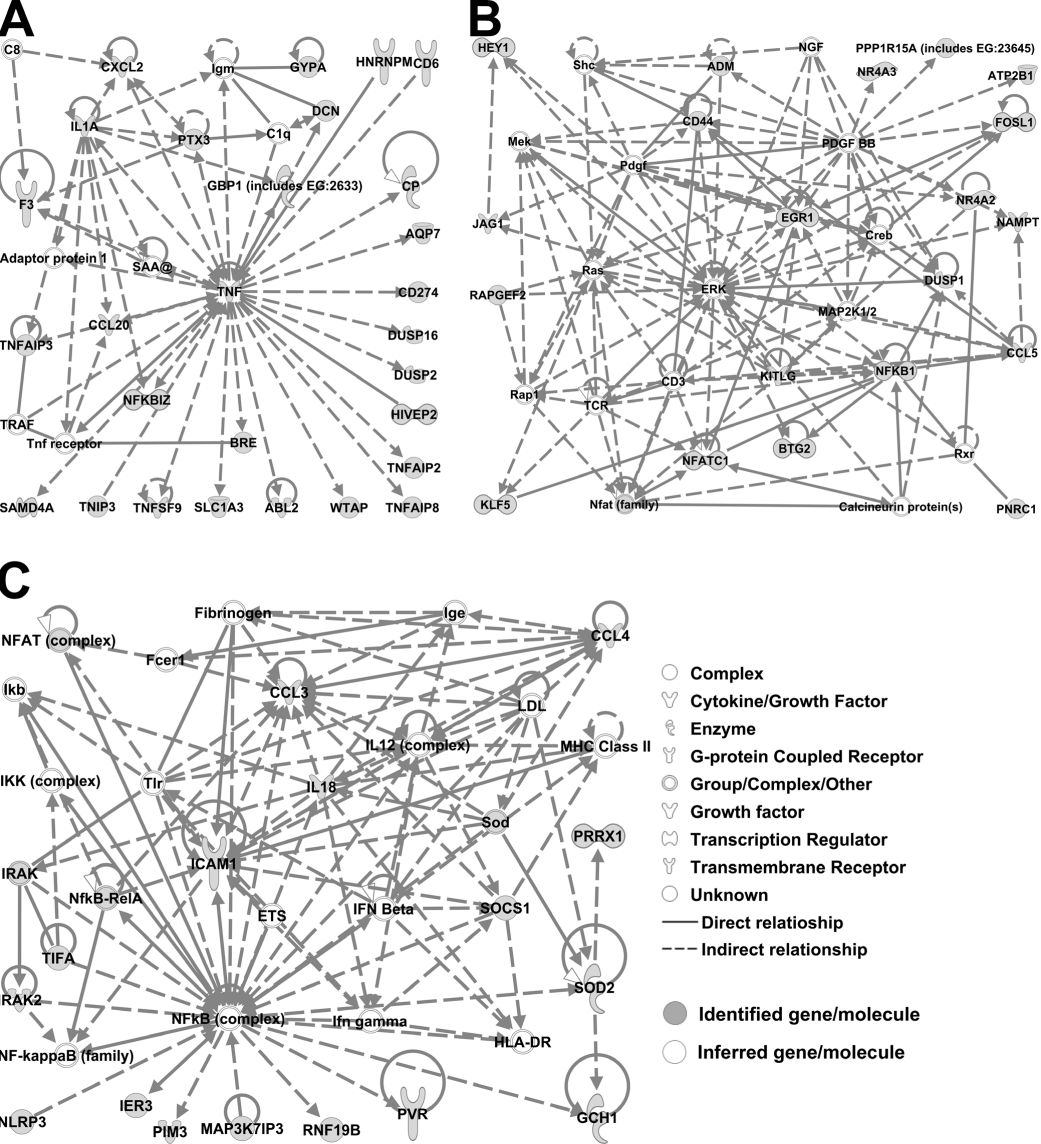
**Networks of genes upregulated on spore-infected cells as determined by Ingenuity analysis**. Genes depicted in gray were identified by the microarray results as differentially expressed. Genes depicted in white are inferred genes. These are genes that should be differentially expressed on the basis of Ingenuity analysis, and although they were expressed, differential expression was not identified by statistical analysis of the microarray results. A) First network, B) Second network, C) Third network. Genes that are not expressed under any conditions were removed from the networks.

Ingenuity formed several well-defined unique networks out of the mapped genes that were induced by spore exposure. Due to computational limitations, each network is limited to 35 genes. Ingenuity ranks networks in order of the consistency of the microarray results with relationships confirmed by prior published results. The three networks with the highest rank are displayed in Figure 2. The first and second networks contained 28 and 20 focus genes (genes identified as up- or down-regulated, marked grey), respectively, and shared similar features. Other network members, shown in white, were inferred by Ingenuity on the basis of literature reports of a connection to a focus gene. These were all manually checked to determine if they were expressed. Usually their expression does not change in the experiment but their expression indicates their participation in signal transduction. Inferred genes that were not expressed were removed. In the first network, TNF-α along with the TNF-α-Induced Proteins 2, 3 and 8 (TNFAIP2, TNFAIP3, TNFAIP8), TNFAIP3 Interacting Protein 3 (TNIP3) and Tumor Necrosis Factor (Ligand) Superfamily, Member 9 (TNFSF9) formed the central hub connecting other up-regulated genes (Figure 2A). The second network contained ERK (extracellular regulated kinase) as a main hub (Figure 2B). This network contained a number of transcription regulators, including BTG2 (BTG family, member 2), EGR1 (early growth response 1), FOSL1 (Fos-like antigen 1), HEY1 (hairy/enhancer-of-split related with YRPW motif 1), NFATC1 (nuclear factor of activated T-cells, cytoplasmic, calcineurin-dependent 1) and NFKB1 (nuclear factor of kappa light polypeptide gene enhancer in B-cells 1). Interestingly, NFκB was the central hub in the third network, connecting genes related to inflammation and the immune response, most notably, NFAT complex, IL-18, CCL3 and CCL4 (Figure 2C).

Ingenuity Pathway Analysis also performed canonical pathway analysis in order to associate differentially regulated genes with known specific biological pathways. The most significant related pathways identified were from cytokine signaling and cellular immune response signaling groups (Table 3). The TREM1 (triggering receptor expressed on myeloid cells 1) signaling was the most significant pathway, followed by bacterial and viral pattern recognition receptor (PRR) pathway. Other pathways and the genes, including Acute Phase Response Signaling pathway, the IL-6 and -10 signaling pathways, and the p38 signaling pathway are shown in Table 3.

**Table 3:** List of most significant canonical pathways overrepresented by upregulated genes in *B. anthracis *infected macrophages.

Ingenuity Canonical Pathways	P-value	Molecules
TREM1 signaling	0.00001	CCL3, ICAM1, IL18, NFKB1, NFKB2, TNF
Role of pattern recognition receptors in recognition of bacteria and viruses	0.00009	CCL5, NFKB1, NFKB2, NLRP3, PTX3, TNF
Acute Phase Response Signaling	0.0002	SOCS1, IL-18, IL-1α, SOD2, CP, NF-κB2, NF-κB1, TNF
IL-6 Signaling	0.0002	SOCS1, IL-18, IL-1α, NF-κB2, NF-κB1, TNF
Hepatic Cholestasis	0.0002	IL-18, IL-1α, ADCY4, NF-κB2, NF-κB1, TNF, IRAK2
p38 MAPK Signaling	0.0002	IL-18, IL-1α, TIFA, DUSP1, TNF, IRAK2
IL-10 Signaling	0.0003	IL-18, IL-1α, NF-κB2, NF-κB1, TNF

Among genes that were down-regulated in spore infected cells, only 46 genes out of 82 were suitable for building networks in Ingenuity. Due to the paucity of genes in this group and to the poor annotation of over half, relatively little could be determined about the function of these down-regulated genes. The poor annotation of over half of these genes indicates the processes involved are poorly understood.

### Analysis of several poorly annotated genes affected in *B. anthracis *infection

Half of the genes upregulated in spore infected cells did not have sufficient annotation to participate in network formation/functional analysis by Ingenuity. Only 39% of downregulated genes were sufficiently annotated, while 61% were unknown to Ingenuity. Thus, more than half of the picture of *B. anthracis *infection remains out of scope. While all differentially expressed genes are listed in the Additional File 1, we performed an analysis of several poorly annotated genes exhibiting the most dramatic changes to provide support that these unknown genes are playing an important role in the biological response to spore infection.

Highly upregulated in spore infected cells, superseding even TNF, was LRRC50 (222068_s_at), leucine rich repeat containing protein. Oncomine analysis identified this gene as being upregulated in lung carcinoma (p = 5E-7), as compared to other cancers in a multicancer study. The assumption behind using Oncomine is that if a poorly annotated gene is found to be expressed exclusively or mainly in lung cancers, then that finding might support a role for the unknown gene in other lung processes. Comparison of the expression of LRRC50 in different lung carcinomas identified it as being upregulated in lung adenocarcinoma (p = 0.002-0.013) and downregulated in squamous cell lung carcinoma (p = 0.006-0.008). Another poorly annotated gene, not expressed under normal conditions and expressed >30SD above noise level in spore infected macrophages was hypothetical protein LOC100130885 (236213_at). Oncomine identified it as being highly upregulated in lung carcinoma in transcriptome profiling of 318 cancer cell lines (p = 8.4E-6). MGC33556, also known as p40, is a hypothetical LOC339541 protein that appeared to be aberrantly expressed, but statistically distinct, in lung carcinoma (p = 1.3E-6 - 1.3E-15). A completely unknown expressed sequence tag 215971_at, upregulated 7.5 times in spore infected macrophages appeared to be strongly downregulated in human papilloma virus positive head and neck cancer (p = 6.4E-6), and in head and neck squamous cell carcinoma (p = 1.7E-6). These results are summarized in Table 4.

**Table 4:** Poorly annotated genes upregulated in spore infected macrophages and their correlation with other conditions.^1^

Affymetrix Probe ID	Gene Name	Gene Description	Ratio	Upregulated in	Downregulated in
222068_s_at	LRRC50	leucine rich repeat containing 50	28.7	Lung Cancer (p = 5E-7) [40]; Lung Adenocarcinoma (p = 0.013-0.002) [41,42]	Squamous Cell Lung Carcinoma (p = 0.006-0.008) [42,43]
236213_at	LOC100130885	hypothetical protein	29.1	Lung Carcinoma (p = 8.4E-6) [44]	
238366_at	MGC33556	hypothetical LOC339541	7.5	Lung Carcinoma (p = 1.3E-6) [40]	Lung carcinoma (p = 1.3E-15) [44]
215971_at		EST	7.5		HPV positive head and neck cancer (p = 6.4E-6) [45] Head and Neck Squamous Cell Carcinoma (p = 1.7E-6) [46]

^1 ^References indicate data sources.

### Common transcription factors are present in genes upregulated by *B. anthracis *spores

**Figure 3:**
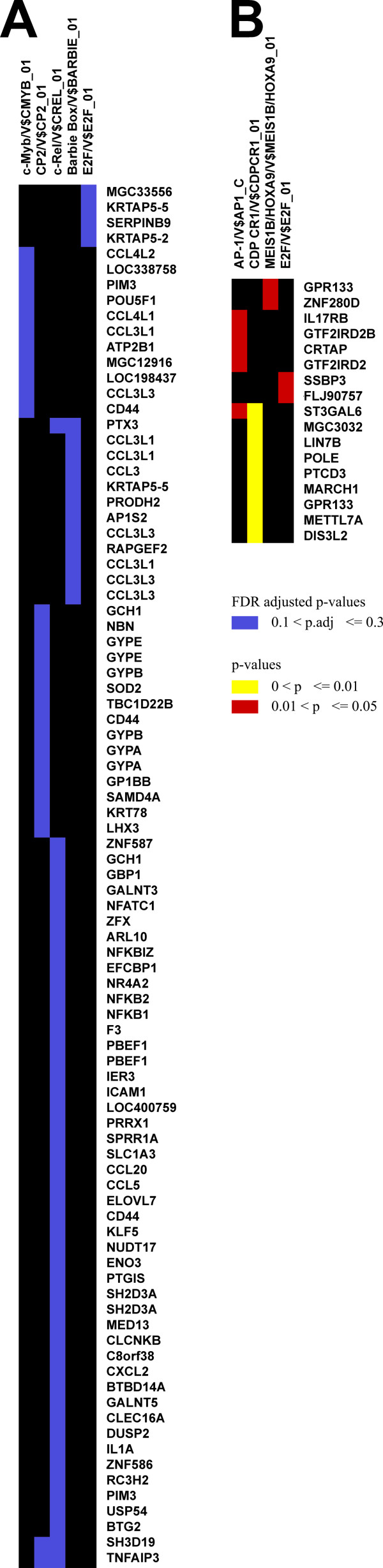
**PAINT clustering of significantly overrepresented TREs**. A) TREs shared by genes upregulated on spore infected cells, filtered by p < 0.05 and FDR<0.3; B) TREs shared by genes downregulated on spore infected cells, filtered by p < 0.05 only.

Analysis of the promoter regions revealed a statistically significant association between the presence of specific TREs, and upregulation of genes by *B. anthracis *spores. The results of promoter analysis filtered by p < 0.05 with the FDR set at <0.3 is shown in Figure 3. The c-Rel TRE, a member of NF-κB family, was associated with induction and was shared by many of the spore-induced genes. It should be noted that the NF-κB-RelA complex was present in the Ingenuity network assembled from those genes (Figure 2C). Other associated TREs were c-Myb, CP2, Barbie Box, E2F and CRE-BP1. Interestingly, the majority of the groups of genes shared only one unique TRE, with few exceptions, like SH3D19 and TNFAIP3 which shared both CP2 and c-Rel TREs (Figure 3A).

Genes downregulated in spore infected cells analyzed with the same stringency did not share common TREs. However, setting less stringent criteria (p < 0.05 only, no FRD restriction) identified AP-1, CDP CR1, MEIS1B and E2F as significant TREs, with CDP CR1 being the most significant, that is, shared by the most number of overexpressed genes (Figure 3B).

### Validation of microarray data by Luminex Protein Array

The results of protein analysis by Luminex assay (Table 5) showed that, for the most part, induction of cytokine mRNA was accompanied by comparable induction of cytokine proteins. For example, TNF-α mRNA induction was induced to a greater extent by spores than almost all of the other cytokines (10.6 fold) and induction of TNF-α protein was the most of any cytokine tested by Luminex (52 fold). IL-6 was also induced strongly by spores both at the level of transcription (27 fold), and translation (24 fold Luminex). Several additional interleukins assayed by microarray and Luminex showed modest, and similar, induction of mRNA (1.4-5.4 fold) and protein (1.4-5.4 fold). Some of the interleukins (IL-2, IL-3, IL-4 and IL-5) were expressed at low levels on both platforms and their fold change was at or near the limits of significance. Interferon-α and γ mRNA were induced ~2-4 fold, although only IFN-γ protein was induced in the current study (5.8 fold). This discrepancy may be because the Affymetrix array contains various IFN-α versions (IFN-α1, IFN-α4, IFN-α16 and others), and the majority of these showed similar ~2-4 fold mRNA increases in spore infected cells. An alternate possibility is that the Luminex analysis was performed on cell supernatants, and the cytokines may be predominantly intracellular. mRNA of the cytokines RANTES (SCYA5, CCL5) and MIP-1α (SCYA3) were also induced ~3 fold and the corresponding proteins were also induced as determined by Luminex (4.7 and 1.7, respectively). There were additional small inducible cytokines (MIP-1β, MIP-1γ, MIP-3α, CCL14 and CCL25) that appeared to be differentially expressed at the level of transcription by spore exposure, but the differences did not reach statistical significance. These cytokines were not included in the Luminex analysis.

**Table 5:** Luminex cytokine assay compared with results from Affymetrix microarray analysis.^1^

Cytokine	Luminex	Affymetrix
IL-1α	2.1	5.4
IL-1β	5.4	2.6
IL-2	Spore only, low expressed	Spore only, low expressed
IL-3	Spore only, low expressed	Not expressed
IL-4	Spore only, low expressed	Not expressed
IL-5	Not expressed	Mock only, low expressed
IL-6	23.7	27.2
IL-7	1.4	0.7
IL-8	0.9	1.5
IL-10	4.6	2.5
IL-12p40	1.4	3.1
IL-12p70	2.0	Not available
IL-13	Spore only, low expressed	1.4
IL-15	Not expressed	1.5
GM-CSF	3.6	1.6
IFN-γ	5.8	3.8
TNF-α	51.6	10.6
Eotaxin	Spore only, low expressed	1.1
MCP-1	2.0	1.7
RANTES	4.7	3.3
MIP-1α	1.7	3.0
IP-10	1.7	Not available
IFN-α	0.8	2.0

^1 ^The data are fold changes over mock.

## Discussion

The alveolar macrophage represents a major defensive mechanism against infection of the lung. Although some details are clear, much is unknown about how these cells respond to pathogens such as *B. anthracis*. In this study a systematic investigation of gene expression of HAM infected by *B. anthracis *spores was undertaken to map out the full response and to identify the full range of genes and pathways involved. The majority of genes that were differentially expressed in response to spore infection were upregulated. Among the upregulated genes, we identified chemokine ligand, apoptosis, and interestingly, keratin filament genes. Central hubs regulating those upregulated genes were TNF-α and NF-κB and their ligands/receptors. Other well known players were IL-1α, IL-18 and others. Many (48) of the spore-induced genes shared c-Rel TREs. C-Rel is a member of the NF-κB family of transcription factors. Other TREs shared in common among spore induced genes were c-Myb, CP2, Barbie Box, E2F and CRE-BP1. In contrast, genes downregulated in cells infected by *B. anthracis *spores did not form well defined ontological groups and networks, neither did they share common transcription regulatory elements, possibly reflecting that down-regulation occurs by different mechanisms than at the transcriptional level.

The method of microarray data analysis utilized here used not only statistical comparisons but also considered biological properties of the data [26] in order to find unbiased results covering the entire genome. By filtering the results using stringent criteria that emphasizes a large change in expression, the specificity of analysis was increased, which identified the most robust "beacons" driving responses to the pathogen. The results provide us with information about the major processes affected. Genes inferred as being present were then checked against the whole dataset to determine that they were expressed. They were removed if expression was not seen in the microarray. This approach removes much of the noise from the system and provides a highly reliable means to identify processes and gene networks. However, as discussed above, all such analyses are limited because the real new knowledge lies in the unannotated or poorly annotated genes, and the pathway and ontologic analyses only confirm that mechanisms studied previously are active in the system under investigation. Some new knowledge also results from showing how these well annotated processes may fit together.

Previous results of gene expression profiling of macrophage responses to *B. anthracis *spores [11,12] have been performed using the murine RAW 264.7 macrophage-like cell line. Although it is difficult to directly correlate those results to primary HAM, several important similarities were observed. In both cases there was induction of genes relating to the immune response, apoptosis, and cytoskeleton organization and biogenesis. Specifically TNFα, IL-1α, colony stimulating factor, IFNγ, and NF-κB were induced in the mouse model [11] and in the current study. On the other hand, other cytokines, for example RANTES (CCL5), is induced by spores only in HAM, and not in RAW 264.7 cells. In these cases, considering that HAM are freshly isolated from normal lung, results with these cells are more likely to reflect those occur during natural infection, than those found with RAW 264.7 cells. Comparison of the current results from microarray analysis with our previously published findings. [13,27] and with additional experimental assays (Table 5) showed high correspondence between gene expression and protein levels. Our previous publication [13] demonstrated *B. anthracis *spore-induced activation of the MAPK signaling pathways, and induction of several cytokines and chemokines. Consistent with that work, the current study demonstrated that the p38 MAPK signaling pathway was overrepresented by genes upregulated in spore infected cells, along with IL-6 and IL-10 associated signaling pathways. Previous results analyzing HAM infected with *B. anthracis *spores for 6 hours at MOI = 1 further confirm the current findings for TNF-α and IL-1β. TNF-α mRNA, induced 11 fold in the current study, was induced 20 fold as determined by RPA. TNF-α protein was induced 74 fold as measured by ELISA. IL-1β mRNA, induced 2.6 fold in the current study, was induced 14 fold in our previous study as measured by RPA. IL-β protein was upregulated by 13-fold as previously determined by ELISA.

IL-6, IL-10, GM-CSF and IFN-γ were also induced 4- to 43-fold in the previous study as measured by the RPA and/or ELISA, and this is consistent with the results for RNA induction as determined in the current study by microarray. The current microarray analysis also identified several additional 2- to 5-fold differentially expressed interleukins and their receptors/binding proteins (IL-1F6, IL-1F7, IL-1F9, IL-1RL2, IL-20, IL-23A, IL-24, IL-29, IL-2RA, IL-32, IL-6ST, IL-7R, IL-9R, IL-11RA, IL-12B, IL-12RB1, IL-15RA, IL-18BP, IL-18R1, IL-19). Two colony stimulating factors, CSF2 and CSF3, were induced 10 and 22 fold, respectively. However, this fold change should be interpreted with caution, due to the high variability of gene expression level for CSF2 and CAF3.

In this study, we performed a static comparison of gene expression in mock- and *B. anthracis *infected HAMs to identify potential hallmarks of inhalational anthrax. However, our results are similar to dynamic changes that occurred in a time course of infection of murine alveolar macrophages by *Aspergillius fumigates *[28]. Nine genes (CCL3, CCL4, CXCL2, EGR1, ICAM1, IL1A, NFATC1, NFKBIZ, TNF) responded dynamically to *A. fumigates *infection and these were also identified as key players in *B. anthracis *induced response. Interestingly, TNF, IL1A, EGR1 and NFκB were the same central players in Ingenuity generated pathways, as in our case (Figure 2). Thus our findngs in the static condition used is simlar to that seen in time course responses of alveolar macrophages to other pathogens.

Inflammation and immune response genes are important in cellular defense responses. This is consistent with our findings, as TREM1 signaling was the most significant pathway identified and was represented by NF-κB, TNF-α and interleukin members. TREM1 belongs to the Immunoglobulin (Ig) family of cell surface recettors and is selectively expressed on blood neutrophils, monocytes and macrophages. It is known that TREM is mediated by a transmembrane adaptor molecule DNAX-activating protein 12 (DAP12), leading to proinflammatory immune responses. The natural ligand for TREM1 is however, unknown. TREM1 signaling is associated with Toll like receptor (TLR) signaling and with a second major class of PRR - the NACHT-LRR receptors (NLR), which recognize intracellular microorganisms. Thus TREM1 acts as an indispensable link connecting (and, possibly, enhancing) signals from both major pathways of pattern recognition- extracellular TLR receptors and the intracellular NLR proteins.

The findings presented here provide additional evidence of the involvement of NLR and TLR signaling in the response to *B. anthracis*. Luminex assays show that IL-1β protein is released from spore exposed HAM. Others have shown that cooperation between MyD88-dependent (TLR) and MyD88-independent (NLR) signaling pathways was required for *B. anthracis *spore mediated IL-lβ induction. Also, both TLR and NLR signaling pathways are important in IL-1β induction and subsequent processing by inflammasome formation [29,30]. This, together with the implication of TREM1 signaling in the HAM response to spores confirms the importance of TLR and NLR in this process.

TNF and NF-κB have long been implicated in inflammation and immune response under various conditions [31-33]. In our system TNF and other members of TNF superfamily were strongly upregulated indicating their involvement in the response to *B. anthracis*. Other genes in the list add to the picture of cellular defense and death triggered by spores. Several interleukins (IL-1α, IL-17RB, IL-18) and TNF have been reported to mediate cell apoptosis and death [34-36], although whether this transcriptional upregulation results in damage to the alveolar macrophage is yet to be determined.

NF-κB is a regulator of TNF and interleukin signaling [37,38]. The transcriptional regulatory element for NF-κB is present in many of the genes overrepresented in cells infected by spores. This may be a key to initiation of the response to *B. anthracis *and a possible target for enhancement of cellular defenses against this pathogen.

This conclusion is also based on our transcriptional regulatory element analysis by PAINT, which assesses genes of interest regardless of their functional classification and considers only TREs shared among them. In this analysis many of the genes upregulated on spore infected cells shared the c-Rel regulatory sequence, a TRE activated by NF-κB. The possibility that this could be a chance finding is low, not only because the stringency was set at p < 0.05, but also because the false discovery rate was set to <0.1. Thus our findings indicated that NF-κB is likely a central regulator in the responses by HAM to *B. anthracis *spores.

*In silico *computer analysis is based on existing information about gene functions and interactions and provides little information about genes that are differentially regulated but poorly annotated. These genes represent undiscovered mechanisms and new knowledge as to processes operating during infection. One possibility for the asymmetry in publications about gene function is that the most likely genes to be discovered are those functioning in all or most cells or tissues. These represent core functions such as apoptosis and cell division. Genes that function in a cell-specific manner are less likely to be discovered, particularly if they occur in cells or tissues that are not heavily investigated. Cell or tissue specificity is likely to be conferred in genes that may be expressed in only a very few cell types, which suggests they should have a higher probability of being poorly annotated. Supporting this is the finding that LRRC50 and three other poorly annotated genes were differentially expressed mainly in lung and head and neck cancers. Our findings thus add to the knowledge about the function of these genes. It is apparent, for example, that these four genes not only function in cancer of the respiratory tract, but also function in the lung response to *B. anthracis*. These findings suggest that to understand the response of human alveolar macrophage to *B. anthracis *will require investigating the functions of these poorly annotated genes (Additional File 1) and how they might be connected to the known pathways.

Another limitation imposed on the current analysis is that Ingenuity limits the number of genes in an individual network to 35. However, individual networks and pathways do not function in isolation. Rather, they are part of a larger system of interacting pathways and genes that make up the response to the pathogen [39]. We observed this in the networks presented in Figure 2, where members of NF-κB and TNF-α interaction appeared in different networks. Therefore, such networks should not be treated as individual entities but rather as a complex network with key genes occupying the "hub" positions.

In summary, the alveolar macrophage shows a complex response to infection by *B. anthracis *spores. Based on the number of poorly annotated genes, only a portion of the response is known. That the expected responders such as NF-κB and TNF were seen gives credence to the importance of these poorly understood genes.

## Conclusion

Taken together, our data provides the first comprehensive description of the response of HAM to *B. anthracis *using microarray. The included analysis is unbiased, as the only consideration for interpretation was the degree and reproducibility of differential expression, as determined by statistical analysis. This minimizes the risk for false positives and provides an objective overview of the cellular response to the pathogen. NF-κB and TNF were the most well characterized hubs governing the response to *B. anthracis *spores and therefore may represent the main targets for enhancing the HAM response to the pathogen. Other less annotated genes represent new, still unexplored targets for intervention at the early stages of inhalational anthrax.

## Competing interests

The authors declare that they have no competing interests.

## Authors' contributions

JPM and KC designed the study and analyzed the data. WW and MD drafted the manuscript. JLB, REH and KMC collected the data and participated to their interpretation. MD performed the statistical analysis. MD, WW and JPM participated to the redaction of the manuscript. All authors read and approved the final manuscript.

## Pre-publication history

The pre-publication history for this paper can be accessed here:

http://www.biomedcentral.com/1471-2334/9/152/prepub

## Supplementary Material

Additional file 1 **List of differentially expressed genes between mock infected and spore infected cells**. Genes overexpressed or only expressed in spore exposed Av - arithmetic mean of gene expression across arrays in a given condition (Mock/Spore). SD - standard deviation of gene expression. P - probability that the expression result is due to background noise, pA - associative p-value for a gene being different between groups. Ratio of average expression levels is presented for reference only, since for uniquely expressed genes the denominator (noise expression level) is meaningless. Groups of genes overexpressed and only expressed in spore infected cells are highlighted. Click here for file

Additional file 2 **Ontological groups overrepresented by genes upregulated in spore- and mock infected HAMs**. The list of ontological groups overrepresented by genes upregulated in spore- and mock infected HAMs. Click here for file
